# What light have resting state fMRI studies shed on cognition and mood in Parkinson’s disease?

**DOI:** 10.1186/2054-7072-1-4

**Published:** 2014-10-29

**Authors:** Sophie YorkWilliams, Kathleen L Poston

**Affiliations:** Department of Neurology and Neurological Sciences, Stanford University, 300 Pasteur Drive, Stanford, CA 94305 USA; Department of Neurosurgery, Stanford University, 300 Pasteur Drive, Stanford, CA 94305 USA; Stanford Neuroscience Institute, 300 Pasteur Drive, Stanford, CA 94305 USA

**Keywords:** Resting state, fMRI, Parkinson’s disease, Non-motor symptoms, Cognition, Memory, Depression

## Abstract

Much remains unknown about non-motor symptoms of Parkinson’s disease (PD), which have variable occurrence, progression, and severity among patients. The existing suite of neuroimaging tools has yielded insight that cannot be garnered by traditional methods such as behavioral and post-mortem assessment. They provide information on brain activity and structure that is invaluable to understanding abnormalities associated with neurodegeneration in PD. Among these tools, functional magnetic resonance imaging (fMRI) is often favored for its safety and spatial resolution. Resting state fMRI research capitalizes on the wealth of information that the brain offers when a person is not performing a motor or cognitive task. It is also a good means to study impaired and heterogeneous populations, such as people with PD. The present article reviews research that applies resting state fMRI to the ongoing hunt for biomarkers of PD non-motor symptoms. Thus far, research in this subfield has focused on two of the most common and significant non-motor symptoms: cognitive impairment and depression. These studies support resting state fMRI as a valid and practical tool for the study of these symptoms, but discrepancies among findings highlight the importance of further research with standardized procedures.

**Electronic supplementary material:**

The online version of this article (doi:10.1186/2054-7072-1-4) contains supplementary material, which is available to authorized users.

## Introduction

A brain at rest is active, and understanding its spontaneous neural activity has in the last decade been recognized as a worthy pursuit [1–5]. Neuroscientists believe that brain cells have the same metabolic system as those in the rest of the body: they require more oxygen and glucose when they are more active. Accordingly, a cluster of brain cells that displays an increase in glucose and/or oxygen use is believed to be more active than when it metabolizes less. With these biological principles in mind, scientists can exploit tools that measure differential glucose or oxygen use in the brain to derive information on brain activity. The least invasive method currently available is functional magnetic resonance imaging (fMRI), which measures blood oxygen level-dependent (BOLD) signals in the brain. In fMRI, the brain is divided into hundreds to thousands of voxels (3D version of pixels), and BOLD signal oscillations within these voxels represent regional changes in brain activity. This makes it a valuable method for researchers to observe brain function, and through experimentation, incrementally discover the underpinnings of human consciousness and cognition [1, 6].

Before scientists appreciated the vast insight that a resting state brain can yield, fMRI was used exclusively for task-based studies, wherein participants perform scanner-compatible assignments while BOLD signals are recorded [6]. These usually involve manual responses to audio/visual cues, which minimizes movement. With creative design, task-based studies can target a wide array of motoric, cognitive, and even psychological functions. However, they are limited by challenging ecological validity and their dependence on participants’ ability to perform the tasks [7, 8]. Resting state (RS) fMRI research provides avenues to discovering brain function, free of these constraints [1, 2, 7]. This is particularly relevant in studies of older participants or impaired populations, such as people with Parkinson’s disease (PD) [9]. In addition, RS fMRI also provides contextually different information than task-based studies. Namely, it reveals brain activity in the absence of attention-demanding external stimuli [10]. To this end, participants are asked to lie still, stay awake, and allow their minds to wander for up to ten consecutive minutes while the resting state data is acquired.

PD is one of many disorders to benefit from RS fMRI research [11]. It is a neurodegenerative disorder with many and heterogeneous symptoms, the most classic being bradykinesia, tremor, and rigidity [12, 13]. While development of Lewy-bodies in the substantia nigra and associated loss of dopaminergic neurons are the definitive pathological marker, the pathophysiologies of non-motor symptoms are not well understood. The most common and impactful of these non-motor symptoms are depression and cognitive impairment [14]. This article reviews research efforts to understand these non-motor symptoms of PD using RS fMRI.

**Figure 1:**
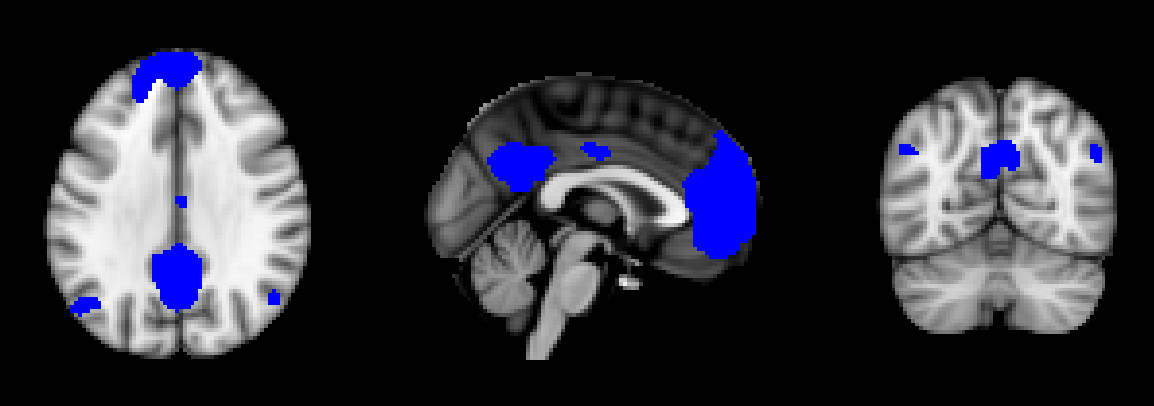
**The default mode network (DMN).** The DMN was identified by applying FSL’s MELODIC independent component analysis software (http://fsl.fmrib.ox.ac.uk/fsl/fslwiki/MELODIC) to the group-level resting-state fMRI data from 15 participants aged 18–30 (see [29] for details of analysis methods). This network includes clusters in the posterior cingulate cortex, precuneus, medial prefrontal cortex, lateral parietal cortex, and the medial temporal lobes (not shown).

Evidence suggests that human cognition results from the dynamic interactions of distributed brain regions acting together as a networks [6, 15]. Using RS fMRI, multiple canonical resting state networks have been described that correspond to critical brain functions including movement, language, episodic memory, and executive function, to name a few [6, 10, 16, 17]. The default mode network (DMN) is currently the most studied network associated with cognition and mood [7]. The term “default-mode” is derived from the observation that specific brain regions are consistently more active when the brain is at rest, mind wandering, than during externally driven tasks [3, 4, 10]. For this reason, it is sometimes called the task-negative or task-free network [4]. The degree to which DMN deactivates during a task depends partially on the type and cognitive load of the task [6, 10, 18, 19]. DMN task-associated deactivation and rest-associated activation have been demonstrated to diverge from normal in some neurodegenerative and neuropsychiatric disorders [20–26]. For example, decreased DMN connectivity has been proposed as a potential biomarker of Alzheimer’s disease because it includes brain regions, such as the posterior cingulate cortex (PCC), precuneus, and the medial temporal lobes, that activate during episodic memory retrieval tasks (Figure 1) [7, 10, 22]. Indeed, the finding that DMN connectivity is reduced in Alzheimer’s disease was one of the first clinical applications of resting state fMRI. More recently, reduced DMN connectivity has been demonstrated in patients with amnestic mild cognitive impairment [27], leading researchers to investigate DMN connectivity changes associated with other patient groups who exhibit amnestic cognitive changes, such as PD. While DMN has been central to almost all RS fMRI research on PD cognitive impairment to-date, it is only one of several reproducible resting state networks critical to human cognition [7, 28]. Other examples of well-studied cognitive RS networks include the executive control network, where connectivity has been associated with executive task performance [17] and the visuospatial network. In addition to DMN, other networks, such as a prefrontal-limbic network, have been the primary focus for depression research [26].

## Review

### Methods

The literature search for pertinent studies was conducted using PubMed and PsycINFO. Varying combinations of the following search terms were used in pursuit of these studies: resting state, resting-state, Parkinson’s, fMRI, functional magnetic, functional connectivity, default-mode, non-motor, cognitive, cognition, depression, depressed, mood, dementia. Relevant peer-reviewed studies published or “in press” in English as of May 2014 were included. Articles were excluded that did not address non-motor symptoms and/or did not use resting state fMRI. In addition, we required that studies include at least one clinical descriptor of the non-motor symptom. For instance, if a study examined the DMN it was included if there was at least one clinical symptom described, such as categorization of participants into cognitive categories or a continuous cognitive variable. Twelve studies met our criteria, and are discussed below.

### RS fMRI analysis techniques

RS fMRI’s relative youth goes hand-in-hand with unknowns surrounding optimal analysis techniques. A range of options exists, which approach the BOLD signal with different goals [4]. In the study of PD, the most commonly used technique for RS fMRI analysis is functional connectivity (FC) [4, 10]. Brain regions are considered functionally connected to the extent that their low frequency BOLD signals fluctuate in sync with one another, regardless of spatial distance [2, 4, 30, 31]. FC analyses are used to understand the brain on a network level, and thus are highly pertinent to RS fMRI research [31–33]. Region of interest (ROI) FC analyses examine the connectivity of a specified area with other specified regions (ROI-to-ROI), or with the whole brain (ROI-to-voxel) [1, 2, 4, 34]. Regions can be defined functionally by selecting an ROI of an area that emerges from other relevant fMRI activation analyses, or anatomically by selecting a brain structure of interest. ROI FC is driven by previous research/hypotheses by nature of its use of pre-defined “seed” regions. This results in hypothesis-driven findings, but also limits the scope of the research to preselected brain regions, therefore ignoring potentially critical findings.

By contrast, a completely data-driven FC approach is independent components analysis (ICA). ICA is a statistical technique that separates a set of signals into independent spatiotemporal components, which are uncorrelated and non-Gaussian. When applied to RS fMRI, ICA disentangles the combined fMRI signal into individual components, which are then defined as networks [30, 31]. Using ICA, one can derive multiple resting state networks, such as DMN or visuospatial network, from a group or individual’s resting state scans. Another example of data-driven resting state fMRI analysis is regional homogeneity (ReHo), which evaluates the 4D activation of each voxel in context of its directly-adjacent voxels [33, 35, 36]. The greater the similarity among neighboring voxels in a scan’s duration, the more regionally homogenous they are. If several touching voxels exhibit near-identical fluctuations in BOLD signal, they represent a cluster of brain activity [33, 35]. The utility of ReHo lies in the (safe) assumption that brain activation is more significant when a cluster is activated at the same time than a single voxel [33, 35, 37]. ReHo does not concern the amplitude of a voxel’s fluctuations; that is the territory of ALFF. Amplitude of low frequency fluctuations (ALFF) analyzes the activation intensity of each voxel in the resting brain by summing the amplitude of its spontaneous fluctuations [38–40]. ICA, ReHo, and ALFF are purely data-driven and typically include the whole brain, which is advantageous given science’s yet-immature picture of its intricacies. Without a priori assumptions limiting the analysis to specific brain regions, these techniques are ideal for discovery of associations not previously detected. However, they can also lack a hypothesis-driven approach and therefore are more prone to false positive errors if the analysis does not include strict correction for multiple comparisons. In addition, these techniques can yield numerous results that can be difficult to deduce and are susceptible to interpretation errors. The relative advantages and limitations of ROI versus data-driven functional connectivity techniques are still a matter of significant debate within the field, and are discussed in detail elsewhere [41, 42].

FC allows researchers to isolate and analyze brain networks, however it does not provide insight into the directionality of the network signals or the relative influence that one region has over another. Effective connectivity models have been developed to address this limitation and allow for study of the dynamic interactions between brain regions. These models aim to understand the neural underpinnings of cognition by using directed links, or edges, to investigate the influence that one neuronal system exerts over another, thereby revealing how cognitive functions arise from interactions within and between distributed brain areas [15, 43]. One such technique that has been used to understand cognitive dysfunction in PD patients is graph theory, which enables connectivity patterns of the whole brain and its connections to be analyzed relative to one another [4, 44, 45]. This method aims to capture both global and local information flow. The theory mathematically represents regions of a network as “nodes,” and direct connections between these regions as “edges”. If two nodes share an edge, then they are directly connected. Nodes that are indirectly connected have longer “path lengths”, because two or more edges are required to connect them. Hubs are nodes that have large influence on information flow among other intra-network nodes. While effective connectivity approaches such as graph theory will likely aid in our understanding of integrated brain functions, these techniques have unresolved methodological issues. Chief among them are the means of node selection and number of nodes to use in analysis, which can significantly alter results [46].

### Cognitive impairment in PD

As with motor symptoms, PD patients are differentially affected by cognitive symptoms – both in terms of symptom subtype and symptom progression. Some degree of cognitive impairment (CI) is almost inevitable in patients who live long enough with PD [46–48], and cognitive deficits can occur early in the disease [45–47]. While levodopa and dopamine agonists can manage motor symptoms for years and even decades, there are limited treatments for CI in PD [49, 50], and nothing is available to prevent or delay cognitive symptoms. In addition, dopamine replacement in PD can have variable effects on cognitive processes, showing improvement or impairment depending in part on the cognitive task being performed [51]. Hence, patients and families often find CI to be a leading symptom impacting quality of life [47]. Further, PD with mild cognitive impairment is a risk factor for PD dementia [52], although the neurobiological underpinnings leading to dementia in PD are complex [53–55].

Advanced imaging techniques, such as RS fMRI, have the potential to enlighten our understanding of PD CI and aid in the development of novel treatment approaches for this debilitating condition. However, there have been several obstacles to RS imaging research inherent to PD CI. For one, there were until recently no standardized guidelines to which researchers could turn for a unified operational definition [56, 57]. Hence, CI meant different things in different studies, making comparison between studies difficult. For the purposes of this review, CI will refer to all forms of cognitive deficit, including the various domains and degrees of severity. We will specify the cognitive domains tested within each study when possible, but this raises the problem of small sample size used in most RS fMRI research. Even when studies include cognitive testing results, they are often too small for comparisons between groups of patients with different types (CI versus dementia) or domains of cognitive impairment. As researchers start to use recommended diagnostic criteria for the domains and severities of CI along with larger sample sizes, the field can hope for more cohesive findings. Lastly, there is uncertainty within the scientific community regarding the effects of dopamine on cognitive RS fMRI connectivity. Despite these obstacles, researchers have begun to explore RS fMRI as a means to better understand cognition in PD patients.

Two studies used an ICA approach to study the relationship between resting state networks and cognition in PD patients. Krajcovicova et al. studied non-demented, non-depressed PD patients and healthy controls [58]. All subjects underwent a structured neuropsychological battery that included assessment of memory, verbal fluency, attention and orientation, speech, and visual–spatial abilities. PD patients underwent all aspects of the study ON their standard dose of PD medications. Applying ICA to resting state data, the authors used a methodologically rigorous combined-group approach to extract DMN network connectivity from all subjects. They did not find any differences in DMN connectivity between PD and controls, nor did any of the behavioral tests correlate with DMN connectivity. This study also used task-based fMRI to examine DMN deactivations during a visuospatial task, which were also not different between groups. However, they found that connectivity within the PCC cluster of the DMN was stronger in patients who were on a higher dose of daily dopamine. They did not find this correlation with disease duration. The authors concluded that non-demented PD patients did not have abnormal DMN connectivity, but noted that dopamine medications may increase DMN connectivity. They postulated that DMN connectivity might be reduced in the dopamine deplete state, and then normalized when patients were medicated. But they cautioned that the hypothesis would need to be studied by comparing the same patients OFF and ON dopamine medications.

Also using ICA, Tessitore et al. included a modest group of cognitively unimpaired, non-depressed PD patients and healthy controls, but the study yielded compelling results [59] (Table 1). Patients were ON their standard anti-parkinsonian medications during scan acquisition and assessment. Although they used a relatively similar ICA technique to extract individual DMN connectivity values, the researchers found different results than Krajcovicova et al. They reported reduced DMN connectivity in PD patients compared to healthy controls, specifically within the right medial temporal lobe and bilateral inferior parietal cortex . The authors clinically assessed three domains of cognition similar to those assessed in the prior study: memory, executive function/attention, and visuospatial ability. Unlike the prior study, they found connectivity changes in DMN regions correlated with individual performance in the cognitive tests. Specifically, the authors found that increased right medial temporal lobe connectivity was associated with better memory performance and increased inferior parietal cortex connectivity was associated with improved visuospatial performance – despite overall memory and visuospatial scores within the normal range. Although the research was conducted before the 2012 diagnostic guidelines for PD-MCI were published, the study’s choice of neuropsychological battery essentially reflected tests recommended in the three domains chosen. The authors found no regions of increased DMN connectivity in the PD group, and did not examine other RS networks. They postulated that one or more of these other networks may be differentially functionally connected in cognitively unimpaired PD patients to compensate for decreased DMN connectivity. Because global grey matter volume, white matter volume, and CSF volume can confound RS fMRI data, the authors confirmed that there were no differences between groups in any of these measures. Unlike Krajcovicova et al., this study suggests that abnormal DMN connectivity associated with memory performance can be identified in PD patients ON dopamine medications, and their findings complement other research where PD dementia has been associated with abnormalities in the medial temporal lobe and parietal cortices [60]. One possible explanation for the discrepancy between these finding and earlier work is differences in methodological approach. Krajcovicova et al. performed the analysis using both groups in the same ICA dataset, which increases the signal-to-noise ratio and is less prone to false positive results. Indeed, Tessitore et al. caution that their results be viewed as preliminary, given the less rigid methodological approach, small sample size, lack of comprehensive neuropsychological battery, and ON dopamine medication scan assessments.

**Table 1:** **Resting state fMRI articles focusing on cognitive impairment in PD**

Authors	Year	Analysis method	Participants (Age in years, mean; SD)	Dopamine medication status	PD Duration (Years, mean; SD)	Controlled for depression	Head motion inclusion criteria; between-group motion comparison
**Krajcovicova et al.**	2011	ICA	18 PD (63.50; 9.07)	ON	PD (5.4; 0.31)	Yes	NR; NR
			18 control (60.89; 6.67)				
**Seibert et al.**	2011	ICA,	19 PD (72; 7)	NR	NR	No	NR; NR
		VBM	18 PRD (70; 8)				
			19 control (76; 9)				
**Rektorova et al.**	2012	Seed FC	18 PD (63.5; 9.07)	ON	PD (4.44; NR)	No	NR; NR
			14 PDD (72.36; 5.88)		PDD (9.64; NR)		
			18 control (60.89; 6.67)				
**Tessitore et al.**	2012	ICA, VBM	16 PD (64.15; 1.64)	ON	PD (5.4; 0.31)	Yes	NR; NR
			16 control (65.5; 6.17)				
**Baggio et al.**	2014	Graph theory	43 PD no-CI (64; 9.8)	ON	PD no-CI (6.1; 4.4)	Yes*	Root mean square <0.3 mm translation or .6° rotation; significant difference found between PD no-CI and controls.
			23 PD CI (66.7; 12.2)		PD CI (9.0; 5.5)		
			36 control (63.4; 10.5)				
**Lebedev et al.**	2014	Graph theory	30 PD (61.67; 9.46)	Naïve	Newly diagnosed, not otherwise specified	No	NR; NA
**Yao et al.**	2014	ICA	12 PD non-visual hallucinations (63.4; 7.4)	ON	PD non-visual hallucinations (8.4; 5.1)	Yes	< 2.5 mm displacement and <2.5° rotation in any direction.
			12 PD visual hallucinations (67.6; 7.4)		PD visual hallucinations (10.0; 3.5)		Did not mention between-group motion comparisons.
			14 control (64.1; 4.0)				

NR: not reported, NA: not applicable. *A significant difference in Beck Depression Inventory scores were found between PD CI and HC that were controlled for in some analyses.

Two studies used an ROI-based analysis to investigate functional connectivity changes in PD patients with dementia. Seibert et al. compared RS FC in three groups: cognitively unimpaired PD patients, healthy controls, and patients with Parkinson-related dementia (PRD) [61]. They did not report the medication state of PD patients during the scan. The third group included not only patients with PD dementia (PDD), but also those who had Dementia with Lewy Bodies. The two groups were combined given the similar underlying pathologies despite distinct clinical syndromes [57, 62].

The authors used an ROI-to-voxel analysis to examine FC using two anatomically-defined seed regions as representative regions within specific resting state networks. They selected a PCC ROI (referred to as the isthmus cingulate) to study the DMN, and the caudate to study corticostriatal networks. The isthmus cingulate yielded no significant or trending FC differences among the three groups. In contrast to Tessitore et al. and similar to Krajocoicova et al., they concluded no DMN abnormalities in PD or PRD; however, it is plausible that other DMN-related seeds would reveal otherwise, since Tessitore et al. found connectivity changes in the medial temporal lobe and parietal DMN regions, but not the PCC. By contrast, the authors found significant corticostriatal network connectivity reductions between the caudate seed and the bilateral superior frontal region, bilateral caudal middle frontal region, and right putamen in PRD patients compared to controls. PD patients without dementia had no significant reductions compared to controls, but exhibited FC patterns that fell between the two other groups. This suggests that a continuum of corticostrial connectivity changes might exist among healthy controls, cognitively normal PD patients, and those with dementia. Studies that also include PD patients with mild cognitive impairment would strengthen this hypothesis, as would longitudinal studies.

As with Seibert et al., Rektorova et al. used FC analyses to seek RS network differences among PD patients with and without dementia, and healthy controls [63]. Participants were assessed ON their usual dose of anti-Parkinsonian medications. The authors used a functionally-derived PCC/precuneus ROI to evaluate DMN alterations, and an anatomically-defined caudate ROI to examine changes in what they termed an extrastriate visual RS network. Similar to Seibert et al., the PCC ROI yielded no significant FC differences across groups at rest. On the other hand, the right caudate had reduced connectivity with both right and left inferior occipital gyri in PDD compared to controls, representing a changed extrastriate visual network. However, the observed areas of FC reduction were in a posterior region that is often susceptible to substantial artifact. That the PDD group had significantly longer disease duration and older age than the other groups was almost unavoidable, since both duration and age are risk factors for development of dementia. The authors attempted to mitigate this confound by using age as a covariate of non-interest, which did not alter the results.

Two studies applied graph theory to RS fMRI data to examine brain networks associated with PD cognitive impairment. Baggio et al. examined network-level changes associated with mild CI in PD while patients were ON medications [44]. Similar but not identical to Tessitore et al., they tested three domains of cognition: attention/executive, visuospatial/visuoperceptual, and declarative memory. Their analysis included PD CI patients, PD no-CI patients, and healthy controls. Results showed long-range connectivity reductions in both PD groups and especially PD CI patients, who had reductions among all major subcortical and cortical areas. Contrarily, these groups displayed an increase in short-range, particularly temporal and prefrontal interlobular connections (again, especially in PD CI). These increased short-range connections were negatively correlated with visuospatial/visuoperceptual and declarative memory performance in CI patients. Both PD groups, but especially CI, showed reduced hub importance and elevated importance of nodes that were less important in HC networks. This reorganization constitutes a shift in PD from more efficient hubs to a reliance on local connectedness. Similar findings have been observed in RS fMRI studies of Alzheimer’s disease [44, 64]. This change may be related to the high metabolic needs of such hubs, which are inherently areas of greater activity [44, 65]. Likely on account of the increased short-range connections, the reduced long-range connections in PD did not affect overall global efficiency or characteristic path length. Differing from results of similar Alzheimer’s disease research, Baggio et al. found that PD CI exhibited increased modularity, which is a measure of how much a network can subdivide into efficient subnetworks (modules) [44], thus yielding a potential biomarker that distinguishes CI in PD from Alzheimer’s disease. Study strengths include the large sample size and sophisticated, data-driven analysis technique. A potential limitation includes a difference in head motion between groups, which has been shown to confound findings in other resting state studies [66]. However, this study overall represents a sizable step forward in PD CI research by identifying a potentially PD-cognition specific network change, which merits further investigation.

Also using graph theory, Lebedev et al. studied a relatively large group of 30 newly diagnosed PD patients, 18 of whom also underwent dopamine transporter imaging [67]. All patients were dopamine-naïve, so scans and cognitive assessments were performed in the OFF state. They applied graph theory analysis using 90 cognitively relevant ROIs to determine nodal strength associated with cognition, again focusing on the memory, visuospatial, and attention/executive domains. Using a Partial Least Squares Regression they estimated the latent components associated with each cognitive domain, and found two cognition-associated components. One displayed better memory performance associated with prefronto-limbic nodes (specifically the orbitofrontal, anterior cingulate, parahippocampal, and temporopolar regions), and the second displayed better executive performance associated with dorsal cortical nodes (dorsolateral prefrontal, frontal, and parietal regions). In the latter, higher caudate dopamine transporter binding was associated with increased nodal strength, suggesting that relative preservation of executive functions is associated with increased dopaminergic influence on dorsal cortical processing.

One study used ICA to investigate visual hallucinations in PD [68]. While the authors did not specifically assess cognitive deficits, we included the findings in our review given the strong association between visual hallucinations and dementia [46]. Yao et al. studied a small group of non-depressed PD subjects with no visual hallucinations, PD with visual hallucinations, and healthy controls (12 in each PD group and 14 controls). The two PD groups were well matched for disease duration, motor severity, and daily levodopa dosage. They only included subjects with a Mini-Mental State Exam score greater than 23 and the average score was matched between the two PD groups; however, this was the only cognitive test reported. All PD patients were scanned ON dopamine medications. The authors applied a dual-regression ICA technique and then masked the data set to only examine regions within the DMN. By only performing the between-group analysis in the DMN-masked regions, they were able to limit the statistical correction for multiple comparisons, but they lost the ability to detect connectivity changes outside of DMN. They found increased DMN connectivity in the right middle frontal gyrus and the bilateral PCC in the PD patients with visual hallucinations compared to those without. However, the severity of hallucinations on the Parkinson Psychosis Rating Scale did not correlate with the relative connectivity strength. The authors also found that both PD groups showed lower DMN connectivity than the controls, specifically in the bilateral medial prefrontal lobe and the PCC/precuneus. The finding that PD patients with visual hallucinations had greater DMN connectivity than those without hallucinations is surprising since hallucinations are often associated with more severe cognitive impairment. However, the authors acknowledge that they were unable to truly assess cognitive function in the sample given the insensitivity of the Mini-Mental State Exam to cognitive impairment in PD. Similar to Tessitore et al., the authors report decreased DMN connectivity the PD groups, however they hypothesize that reduced DMN connectivity may be attributable to PD in general rather than secondary to cognitive deterioration.

While reduced DMN connectivity has been associated with episodic memory changes in patients with amnestic mild cognitive impairment and Alzheimer’s disease, studies in PD patients thus far suggest that reduced DMN connectivity is not associated with PD cognitive impairment. There are several factors that might explain these findings, however. First, it is interesting that studies focusing on the PCC did not find an association between DMN connectivity and PD cognition; by contrast, the one study that found a relationship between PD cognition and DMN only identified reduced connectivity in the medial temporal and posterior parietal DMN regions. In addition, no studies have examined DMN changes specifically in PD patients with and without amnestic CI, which is important considering the relationship between DMN connectivity and episodic memory impairment in Alzheimer’s disease. Finally, while most studies controlled for depression [44, 58, 59, 68], a few did not [61, 63, 67] (Table 1). Given the high prevalence of depression in PD [69] and prior studies suggesting DMN connectivity changes in depressed patients [70], it is critical that future studies exploring DMN changes in PD patients control for this potential confound.

The studies presented in this review highlight the importance of examining networks other than DMN when investigating PD cognition. Specifically, inferior parietal connectivity changes associated with visuospatial performance found by Tessitore et al. and PD dementia related connectivity changes between the caudate and frontal cortex found by Seibert et al. suggest cognitive networks other than DMN might be abnormal in PD cognitive impairment. This is in line with findings that non-DMN networks are impaired in other dementia syndromes, such as behavioral variant frontotemporal dementia [48, 71]. In addition, functional imaging at rest using ^18^F-fluorodeoxyglucose positron emission tomography has shown an abnormal network of glucose metabolism in the pre- supplementary motor area, prefrontal cortex, and parietal association regions associated with PD cognitive impairment [12], suggesting that cortical regions in non-DMN networks could contribute to PD associated cognitive impairment. Whether non-DMN networks such as the visuospatial network or executive control network, for example, are abnormal in PD has yet to be explored. Considering that PD cognitive impairment often occurs in multiple domains, is it also likely that different networks are related to different types of cognitive impairment, which is supported by the findings of Lebedev et al. As previously mentioned, this matter can only be resolved using larger studies that include a thorough assessment of all cognitive domains.

Several studies examined corticostriatal networks associated with cognition in PD. Specifically, the findings by Lebedov et al. suggest a distinction between dopamine medicated executive performance associated with a dorsal cortical network and a non-dopamine dependent network associated with memory. The findings of both Rektorova et al. and Seibert et al. demonstrated subcortical-cortical connectivity reductions associated with PD dementia, although one found caudate-frontal cortex deficits and the other caudate-occipital cortex deficits. Using task-based fMRI other studies have hypothesized a distinction between frontostriatal PD cognitive impairment, which is milder and dopamine dependent, and posterior (primarily parietal and temporal) PD dementia [72]. Overall, the studies discussed in this review support the idea of dopamine dependent and non-dopamine dependent cognitive networks in PD, and they suggest that RS fMRI could be a useful tool in expanding our understanding of how corticostriatal connections are associated with different cognitive deficits. However, with one exception [67] cognitive RS fMRI studies in PD patients thus far have been performed ON medications and therefore lack a study design to directly assess aspects of cognitive RS networks that might be sensitive to dopamine. Nevertheless, Krajcovicova et al.’s findings that DMN connectivity correlated with daily dopamine dosage, but not disease duration, suggests that cognitive RS networks can indeed be altered by dopamine. This is not surprising since several studies have already identified dopaminergic changes in PD motor resting state networks [73, 74], including dopaminergic connectivity changes between the basal ganglia network, the medial prefrontal cortex and the precuneus [75]. It is conceivable that dopamine could ‘normalize’ certain RS networks in PD patients, but further perturb others. Future cognitively focused studies in PD patients OFF and ON dopamine will be an important next step given the dichotomous clinical cognitive response to dopamine in PD.

Finally, the studies applying graph theory by both Baggio et al. and Lebedev et al. suggest techniques that detect strength and directionality of connectivity, rather than simply network identification, might provide more accurate understanding of PD cognitive circuitry. This is especially important considering current interest in using brain modulation techniques, such as deep brain stimulation, to treat cognitive impairment [76]. It is plausible that applying these effective connectivity techniques to RS fMRI could assist in identifying ‘hubs’ of cognitive control in PD that could be the target of direct stimulation interventions.

### Depression in PD

Depression is clinically relevant in approximately 35% of people with PD and constitutes a major impact on patients’ quality of life [9, 69, 77]. Non-PD depression fMRI research has yielded abnormalities in two RS networks: the DMN and a prefrontal-limbic network that includes the prefrontal cortex, anterior cingulate cortex, amygdala, palliostriatum, and the medial thalamus [26]. Within the DMN, findings include both increased and decreased RS functional connectivity, even within the same patients, suggesting a shift in network connectivity rather than overall network suppression [70]. Within the prefrontal -limbic network, studies have shown abnormal functional connectivity specifically between the subgenual anterior cingulate cortex and other limbic structures [78]. This is consistent with ^18^F-fluorodeoxyglucose positron emission tomography studies showing the subgenual cingulate region is metabolically overactive in treatment-resistant depression, an insight that has led to exploration of subgenual deep brain stimulation therapy for depression [79]. We will discuss the five RS fMRI studies that explore connectivity changes associated with depression in PD. Overall, this research presents evidence of a disrupted prefrontal-limbic network in depressed PD patients similar to that in non-PD depression (Table 2).

**Table 2:** **Resting state fMRI articles focusing on depression in PD**

Authors	Year	Analysis method	Participants (Age in years, mean; SD)	Dopamine medication status	PD Duration (Years, mean; SD)	Head motion inclusion criteria; between-group motion comparison
**Skidmore et al.**	2011	ALFF	15 PD (62; 9)	OFF	NR	<1.5% coefficient of variance; NA
**Wen et al.**	2013	ALFF	17 PD depressed (64.4; 13.4)	OFF	PD depressed (6.4; 5.4)	<2 mm displacement each in translational or rotational and <2° during whole scan; found no significant differences between groups.
			16 PD non-depressed (60.7; 18.7)		PD non-depressed (5.6; 7.4)	
			21 HC (55.4; 16.4)			
**Luo et al.**	2014	ALFF	29 PD depressed (51.46; 8.21)	Naïve	PD depressed (1.98; 1.64)	<1.5 mm and <1.5° displacement in any direction; found no significant differences between groups.
			30 PD non-depressed (53.64; 10.18)		PD non-depressed (2.12; 1.30)	
			30 HC (51.9; 7.7)			
**Luo et al.**	2014	Seed FC	52 PD (52.28; 9.41)	Naïve	PD (1.94; 1.47)	<1.5 mm and <1.5° displacement in any direction; NR.
			52 HC (51.17; 9.23)			
**Sheng et al.**	2014	ReHo	20 PD depressed (55.9; 7.4)	OFF	PD depressed (3.4; 1.7)	<2 mm or 2°displacement in any direction; no significant differences between groups.
			21 PD non-depressed (57.3; 6.1)		PD non-depressed (4.0; 2.4)	
			25 HC (56.7; 5.3)			

NR: not reported, NA: not applicable.

Luo et al. published two studies assessing early-stage PD patients with and without depression, along with healthy controls [40, 80]. They included only drug-naïve patients, thereby controlling for the potentially cofounding effects of chronic dopamine replacement therapy on clinical symptoms and resting state brain activity. In one study [40], they found that depressed PD patients had higher ALFF in the left orbitofrontal cortex than healthy controls and non-depressed PD patients. This activity correlated positively with Hamilton Depression scores in the depressed group, which was attributed to an increased orbitofrontal effort to control the limbic system. The authors then chose 19 anatomical ROIs in the prefrontal and limbic regions for an ROI-to-voxel FC analysis, which revealed that depressed PD patients had uniquely reduced connectivity between the left orbitofrontal cortex and right insula. This prefrontal-limbic disturbance was unrelated to motor symptom severity, which was equal across the PD groups. The authors also found that while both PD groups had reduced connectivity between the putamen and the amygdala, hippocampus, and other mesolimbic areas, depressed patients exhibited additional reduced connectivity between the putamen and the left middle temporal gyrus. Their other published study [80] reported complementary findings and used the Non-Motor Symptoms Scale. They found that mesolimbic-striatal connectivity correlated significantly with the Non-Motor Symptoms Scale scores. Specifically, decreased right amygdala connectivity with the putamen was associated with higher (more severe) global Non-Motor Symptoms Scale score and mood subscore. No other specific non-motor subscores correlated with connectivity. Luo et al. are the only group thus far to report significant changes that distinguish depressed PD from both non-depressed PD and healthy controls.

Using ReHo analysis, Sheng et al. found regional synchronization abnormalities associated with depression in PD while patients were OFF dopamine medications [35]. They then used regions with group differences in ReHo for ROI-to-voxel FC analyses. Their cohort was not older than Luo et al.’s, but had longer disease duration. Compared to non-depressed PD patients, they found significant ReHo decreases in the left amygdala and bilateral lingual gyrus, and ReHo increases in the left middle frontal gyrus and right inferior frontal gyrus in the depressed group. These increases are consistent with mood literature, where research has demonstrated resting state hyperactivity in these regions [35, 81]. The ROI-to-voxel FC analysis revealed several differences among groups, which are listed in Table 3. Notably, depressed patients had decreased left amygdala-prefrontal gyrus connectivity than non-depressed patients, again in line with general depression research [34, 35]. However, the authors did not find significant connectivity differences between depressed PD patients and healthy controls.

**Table 3:** **Sheng et al.’s significant findings – functional connectivity changes in depressed versus non-depressed PD**

Connectivity Decreases:	
	Amygdala – prefrontal gyrus
	Right inferior frontal gyrus & left cerebellum
	Right inferior frontal gyrus & right cuneus
	Bilateral lingual gyrus & right superior frontal gyrus
	Bilateral lingual cortex & left middle frontal gyrus
**Connectivity Increases:**	
	Right inferior frontal gyrus & right insula
	Left middle frontal gyrus & right parietal gyrus
	Right inferior frontal gyrus and left lingual gyrus
	Bilateral lingual gyrus & bilateral median cingulate gyrus

In addition to Luo et al., two studies applied ALFF to RS fMRI to study connectivity in PD depression. Skidmore et al. studied depressed and non-depressed PD patients while OFF medications [38]. They found significantly increased ALFF in the right subgenual cingulate cortex of depressed patients, which correlated with higher (worse) Hamilton Depression scores. The authors also found that patients with high caregiver-rated apathy exhibited increased right middle frontal gyrus ALFF and decreased left supplementary motor region ALFF at rest, in addition to the increased ALFF in the right orbitofrontal cortex (which had slightly different peak coordinates from the subgenual-focused depression marker). With this Skidmore et al. went a step beyond the other groups by finding an RS fMRI biomarker specifically for caregiver-rated apathy. They concluded that there are distinct abnormal networks associated with PD depression and PD apathy, which is interesting in light of evidence that selective serotonin reuptake inhibitor treatment for depression might increase apathy in PD patients [82]. This finding also has important implications in the development of deep brain stimulation for PD mood disturbances and suggests that different targets could differentially affect depression and apathy [79].

Wen et al. studied depressed and non-depressed PD patients OFF anti-parkinsonian medications, and healthy controls [83]. The authors found that compared to non-depressed PD patients, those with depression had decreased ALFF in the right dorsolateral-PFC, right ventrolateral-PFC, and rostral anterior cingulate cortex – all components of a prefrontal-limbic network. The dorsolateral-PFC is generally credited for down-regulating affective responses to negative situations, and its dysfunction is widely noted in depression research. The authors also found increased ALFF in the right side of the cerebellum in the depressed PD group. Despite these prefrontal/cingulate discrepancies with the other studies, they found higher ALFF in the dorsolateral-PFC was associated with higher (worse) Hamilton Depression scores. Wen et al. did not administer assessments of mood or cognitive function in their control group, which is a limitation of the study.

Some consistent RS fMRI findings have emerged between PD and non-PD depression research. Both studies by Luo et al. suggest that PD patients with depression have decreased connectivity within the prefrontal-limbic cortex previously identified in non-PD depression, and specifically between the amygdala and putamen. Two other studies found a relationship between increased ALFF in the orbitofrontal cortex and clinical measures, including caregiver-rated apathy [38] and patient-rated depression [40]. Wen et al. also found a positive correlation between increased ALFF and patient-rated depression, but only in the dorsolateral-PFC. Other findings by Wen et al. diverge from PD and non-PD depression research, such as PD depression-related *decreases* in prefrontal-limbic ALFF; the authors did not explain this seemingly paradoxical relationship (i.e., overall decreased dorsolateral-PFC ALFF in PD depression, but a positive correlation between dorsolateral-PFC ALFF and depression scores). Finally, it is unclear if connectivity changes in PD-depression differ from those in non-PD depression because thus far RS fMRI studies have not directly compared these two groups. While DMN connectivity changes have been implicated in non-PD depression, this network was not specifically assessed in the current PD RS fMRI depression studies.

In general, the studies using RS fMRI to investigate depression in PD control for several important confounds. For instance, all studies concluded that no relationship existed between depression-associated connectivity changes and motor severity. Unlike the previously discussed studies on PD cognitive impairment, all studies in depression were either conducted in the OFF medication state or in dopamine naïve patients and thereby control for dopamine-associated changes in connectivity. Finally, all studies report head motion exclusion criteria, and all but one study specifies that there were no between-group differences in movement (Table 2).

## Conclusions

Resting state fMRI has emerged as an important tool in the study of human cognition and emotion, and recently has been applied to patients with PD in an effort to enlighten our understanding of these critical non-motor symptoms. In general, these studies support RS fMRI’s potential to be a valid and practical tool for the study of non-motor symptoms in people with PD. Researchers acquired high-quality RS fMRI data without signal degradation from tremor or dyskinesias in both OFF and ON PD medication states. Studies of PD cognition have suggested that functional connectivity between different brain networks could be associated with impairment in different cognitive domains. Studies of PD depression have suggested functional connectivity changes in PD are similar to those in non-PD depression, while a direct comparison between these two groups has yet to be explored. Despite these steps forward, discrepancies among results urge the field to replicate studies with careful attention to several methodological issues. The role of dopamine medication on non-motor RS networks needs to be better explored, particularly given the varying role these drugs play in non-motor symptomatology and the clear association between dopamine and RS motor network changes demonstrated in prior studies [73, 74]. Technical issues present another barrier to cross-study comparability. Movement difference between groups, and not simply total head movement, has recently been shown to influence resting state results and needs to be controlled carefully in PD patients (Tables 1 and 2) [66]. Larger studies in well-defined patient groups according to published diagnostic criteria, along with detailed demographic and clinical data, are necessary to interpret and compare findings associated with patient subgroups, such as PD-CI versus dementia and domain-specific impairments. Finally, longitudinal studies are required to determine if any of these findings can be used to predict future changes in PD cognition or mood. Studies that attend to these issues could yield early clues of individual patient risk for developing PD non-motor symptoms including cognitive impairment and depression, and such biomarkers are the brass ring of PD research.

## Authors’ original submitted files for images

Below are the links to the authors’ original submitted files for images. Authors’ original file for figure 1 Authors’ original file for figure 2 Authors’ original file for figure 3

## Abbreviations

ALFF: Amplitude of low frequency fluctuations
BOLD: Blood oxygen level-dependent
CI: Cognitive impairment
DMN: Default mode network
FC: Functional connectivity
fMRI: Functional magnetic resonance imaging
ICA: Independent components analysis
MCI: Mild cognitive impairment
NMSS: Non-Motor Symptoms Scale
PCC: Posterior cingulate cortex
PD: Parkinson’s disease
PDD: PD dementia
PFC: Prefrontal cortex
PRD: Parkinson-related dementia
ReHo: Regional homogeneity
ROI: Region of interest
RS: Resting state.
