# Supplementary figures and images for: What light have resting state fMRI studies shed on cognition and mood in Parkinson’s disease?
Source: J Clin Mov Disord. 2014 Oct 29;1:4. doi: 10.1186/2054-7072-1-4 (PMC4677732; doi:10.1186/2054-7072-1-4)

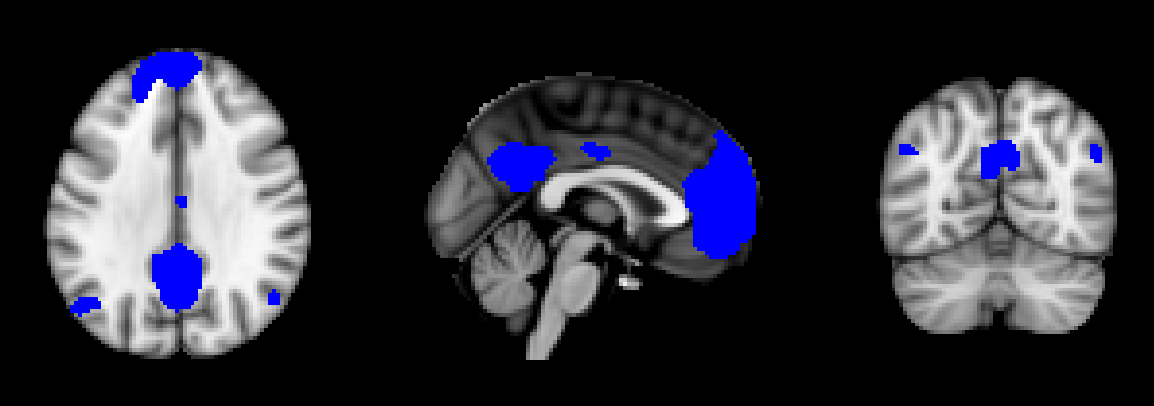

Supplement: Supplementary file 1 — Authors’ original file for figure 1 [file 40734_2014_4_MOESM1_ESM.png]

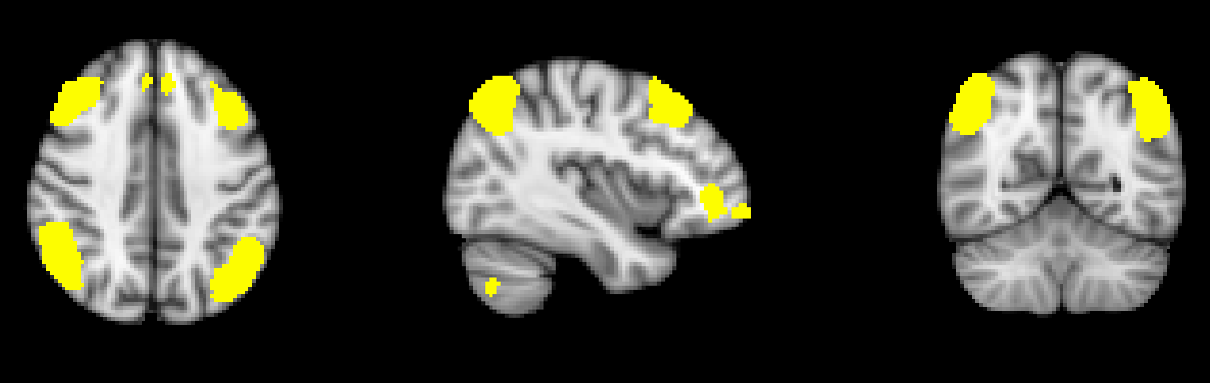

Supplement: Supplementary file 2 — Authors’ original file for figure 2 [file 40734_2014_4_MOESM2_ESM.png]

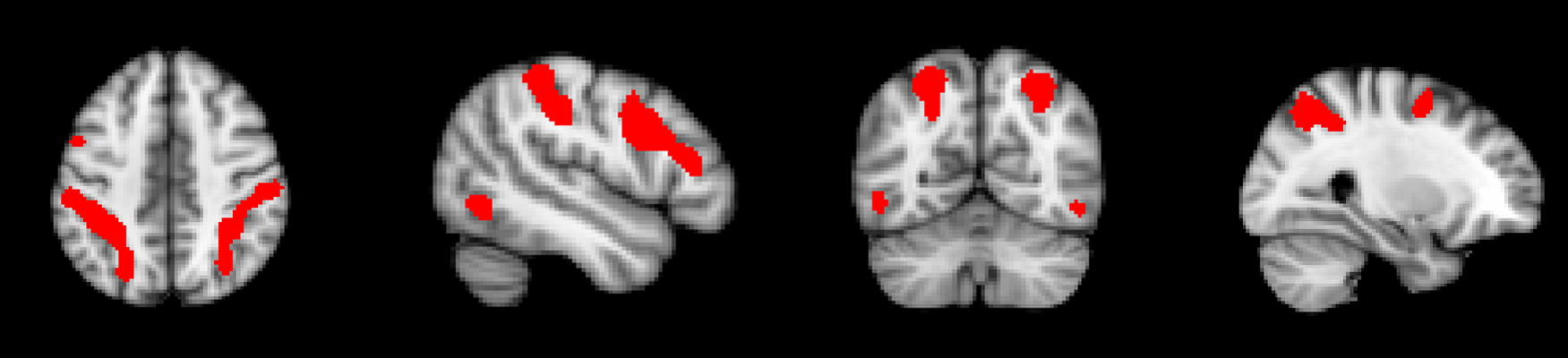

Supplement: Supplementary file 3 — Authors’ original file for figure 3 [file 40734_2014_4_MOESM3_ESM.png]
